# Mini-Cog to Predict Postoperative Delirium in Patients Who Underwent Transurethral Resection of Bladder Tumor While Awake

**DOI:** 10.5152/tud.2022.21312

**Published:** 2022-03-01

**Authors:** Shugo Yajima, Yasukazu Nakanishi, Shunya Matsumoto, Naoya Ookubo, Kenji Tanabe, Madoka Kataoka, Hitoshi Masuda

**Affiliations:** National Cancer Center Hospital East, Chiba, Japan

**Keywords:** TURBT, delirium, Mini-Cog, screening tool, cognitive impairment

## Abstract

**Objective:**

In the postoperative management of transurethral resection of bladder tumor, attention should be paid to the appearance of delirium. Recently, the mini-cognitive assessment instrument (Mini-Cog) has been validated as a screening tool for cognitive impairment. We assessed whether positive preoperative cognitive impairment screening by Mini-Cog is associated with the occurrence of postoperative delirium.

**Material and Methods:**

In this study, consecutive patients who underwent transurethral resection of bladder tumor while awake and were cognitively screened preoperatively using the Mini-Cog test at our institution were retrospectively analyzed. The relationship between the Mini-Cog test and clinical variables was examined. Univariate and multivariate analyses were carried out to determine the risk factors for the occurrence of postoperative delirium.

**Results:**

Of the 193 included patients, 37 (19%) patients had probable cognitive impairment (Mini-Cog scores < 3). There were significant differences in patients’ age (*P* < .001), Eastern Cooperative Oncology Group-physical status (*P* = .01), decline in instrumental activities of daily living from baseline (*P* = .03), preoperative diagnosis of dementia (*P* < .001), and use of benzodiazepine (*P* = .03) between the Mini-Cog score ≥ 3 group and the Mini-Cog score < 3 group. Multivariate analysis demonstrated that a Mini-Cog score < 3 (odds ratio = 6.8, *P* < .001) and instrumental activities of daily living decline (odds ratio = 3.0, *P* = .02) were independent risk factors for the occurrence of postoperative delirium.

**Conclusion:**

Screening of patients for cognitive function using the Mini-Cog test before transurethral resection of bladder tumor may allow for better identification of patients at risk of postoperative delirium.

## Main Points

- We demonstrated the clinical significance of a Mini-Cog score < 3 for predicting postoperative delirium after transurethral resection of bladder tumor performed under awake spinal anesthesia.
- Cognitive function screening using the Mini-Cog test before surgery may help identify patients at risk of postoperative delirium.
- The Mini-Cog test is a simple screening tool with only 2 components (a delayed, 3-word recall task and a clock drawing test). It may indicate a preoperative cognitive decline in patients who have not yet been diagnosed with dementia.

## Introduction

Bladder cancer is one of the most common cancers, and its incidence increases with age: the median age at diagnosis is 73 years. Among male patients older than 80 years, bladder cancer is the fourth leading cause of cancer-related death.^1^ Although there are various investigative modalities to aid in the diagnosis of bladder cancer, transurethral resection of bladder tumor (TURBT) is the mainstay for the diagnosis and treatment of bladder cancer. In the postoperative management of TURBT, special attention should be paid to the physiological changes associated with aging and comorbidities, especially in elderly patients.

Baseline cognitive impairment is one of the most important risk factors for the development of postoperative delirium in the elderly.^2^ Delirium is often a problem in the intensive care unit or after surgery performed under general anesthesia, but postoperative delirium can also occur after surgery under spinal anesthesia while awake. Delirium after TURBT can lead to inappropriate self-extractions of the Foley catheter. Traumatic Foley catheter self-extractions can cause permanent urological complications, increase hospital length of stay, decrease patient satisfaction grades, and increase catheter-associated urinary tract infections. Rarely, they can cause life-threatening hematuria which may require pelvic arterial embolization to control.^3^

Recently, the mini-cognitive assessment instrument (Mini-Cog) has been validated as a screening tool for dementia^4^ and cognitive impairment^5^ and is feasible for use during the preoperative clinic valuation. This test consists of 2 components: a delayed, 3-word recall task and a clock drawing test.^6^ Although the preoperative screening of cognitive function using the Mini-Cog test is useful in predicting the occurrence of delirium after general anesthesia,^7,8^ the association between the Mini-Cog test and delirium after surgery under awake conditions has not been evaluated.

In this study, we assessed whether positive preoperative cognitive impairment screening by the Mini-Cog test is correlated with the occurrence of postoperative delirium in patients who underwent TURBT while awake.

## Materials and Methods

### Patient Population and Study Design

Ethical approval was obtained from National Cancer Center Hospital Institutional Review Board (approval number: 2018-159). We retrospectively reviewed the medical records of patients who underwent TURBT under spinal anesthesia in our institution between January 2020 and July 2021. Cases in which the Mini-Cog test was not completed in the preoperative clinic before the scheduled surgery were excluded from this study. For patients who underwent multiple surgeries within the time period, only data from the first TURBT were used, and data from the second and subsequent surgeries were excluded.

The following patient variables were collected: gender, age, Eastern Cooperative Oncology Group-physical status (ECOG-PS), Charlson comorbidity index (CCI), instrumental activities of daily living (IADL), any prior diagnosis of dementia, diagnosis of depression, preoperative medications, operative time for TURBT, presence of TURBT in one piece, tumor location, tumor size, number of tumors, tumor stage, intraoperative hypotension, intraoperative bradycardia, perioperative shivering, intraoperative use of ephedrine, intraoperative use of atropine, surgical/postoperative complications, and length of hospital stay.

### Study Procedures

**Figure 1.**
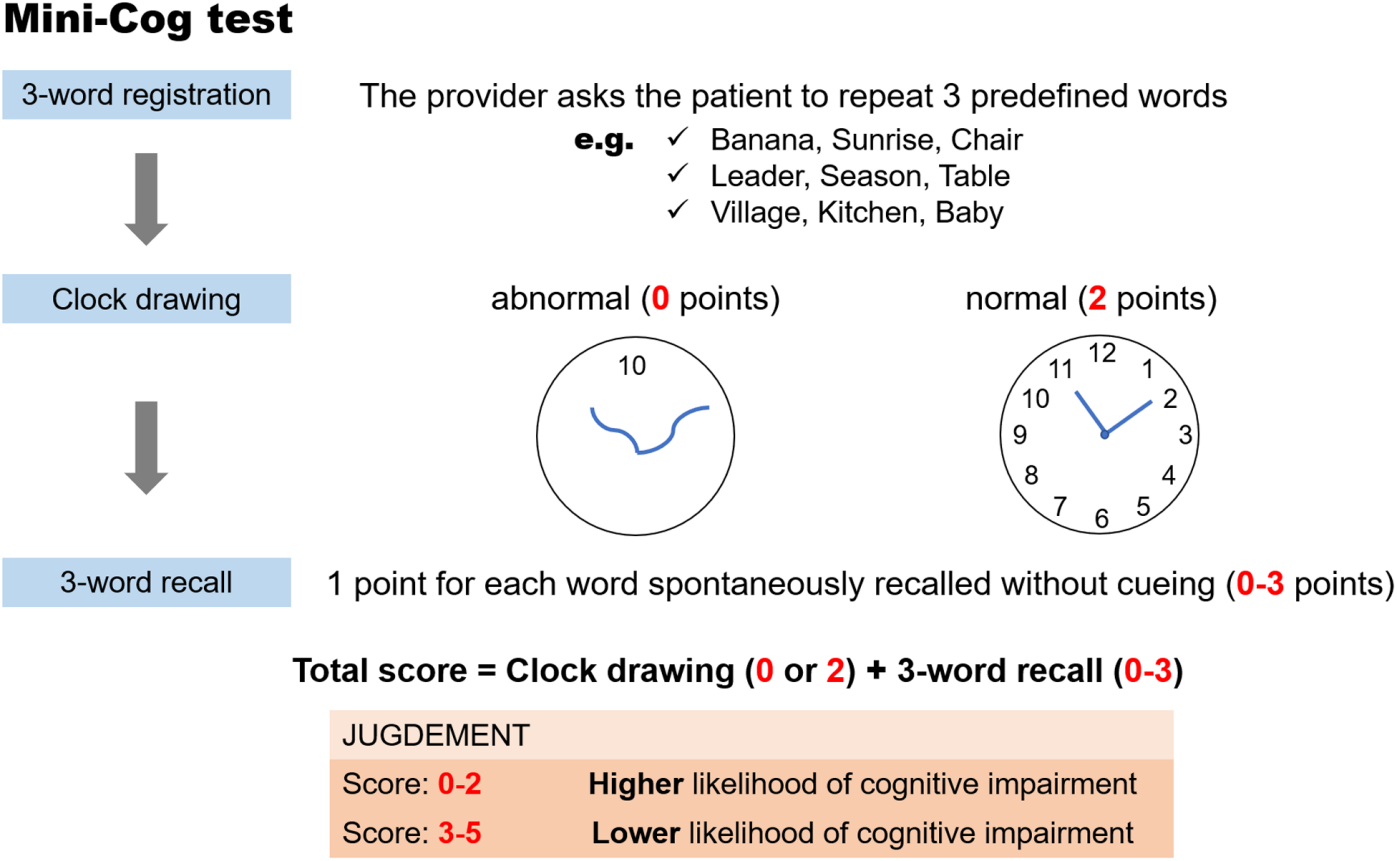
Scoring algorithm of the Mini-Cog test.

Eligible patients underwent the Mini-Cog screening test in the preoperative clinic. Briefly, the Mini-Cog is scored in 2 parts: (1) 3-item recall and (2) clock drawing.^4,6^ The Mini-Cog test starts with a list of 3 predefined words, which the provider asks the patient to repeat. The patient is then asked to draw a clock and, finally, to recall the predefined words. The maximum test score is 5, and points are subtracted for each error in word recall (1 point for each) or for abnormal clock drawing (2 points). Probable cognitive impairment is defined as a Mini-Cog score < 3 (Figure 1). Abnormal clock drawing is defined for any of the following reasons: refusal to draw the clock, the patient takes longer than 3 minutes, and incorrect drawing of the clock.^4,6^ The Mini-Cog test has been validated in community-based populations with high sensitivity and specificity for cognitive impairment and shows good interrater reliability.^9^

In this study, IADL was assessed by an IADL subscale consisting of 5 questions in males and 8 questions in females. Patients were asked whether they could do a particular activity: using public transportation, shopping for daily necessities, preparing meals, paying bills, managing a bank account, and so on. Females were asked additional questions about whether they could perform activities related to preparing a meal, laundry, and cleaning. The total score of the IADL subscale ranged from 0 (worst) to 5 (best) in males and 0 (worst) to 8 (best) in females. We defined patients whose IADL scores declined by 1 or more points from the best score (males: 5 and females: 8) as patients with “IADL decline.”^10,11^

Charlson comorbidity index was categorized as CCI 0-1 vs CCI ≥ 2. Eastern Cooperative Oncology Group-physical status was categorized as ECOG-PS 0-1 versus ECOG-PS ≥ 2.^12,13^

Transurethral Resection of Bladder Tumor was performed in the operating room. Intraoperative hypotension was defined as systolic blood pressure of < 80% of the baseline value; bradycardia was defined as heart rate of < 50 beats per minute.^14^ A half-day of bed rest was prescribed for patients after surgery. The presence of postoperative delirium was diagnosed based on the American Psychiatric Association’s *Diagnostic and Statistical Manual of Mental Disorders* (*DSM*)-5^15^ as a disturbance in attention and awareness that develops over a short period of time (hours to a few days) and fluctuates in severity during the day. 

## Statistical Analysis

The relationship between the Mini-Cog test and clinical variables was examined. In addition, we evaluated the association between the Mini-Cog test and the occurrence of postoperative delirium. 

We performed the Mann–Whitney *U* test for continuous variables and the chi-square test or Fisher’s exact probability test for categorical variables. Univariate and multivariate logistic regression analyses were used to evaluate the predictive factors for the risk of the occurrence of postoperative delirium. Reduced models were generated by backward elimination of the variable with the highest *P* value from each iteration. Risks are presented as an odds ratio (OR) and 95% CI. All *P* values of < .05 (two-sided) were considered statistically significant. All statistical analyses were performed using JMP 13 software (SAS Institute Inc., Cary, NC, USA).

## Results

### Study Population

Among the 284 consecutive cases who underwent TURBT in our department between January 2020 and July 2021, the following cases were excluded: 2 cases who underwent only transurethral coagulation, 8 cases who underwent TURBT under general anesthesia, 43 cases who were duplicate TURBT surgeries/duplicate TURBT records of the same patient, and 38 cases for whom the Mini-Cog test was not completed in the preoperative clinic before the scheduled surgery. Thus, we analyzed the remaining 193 patients, with a median age of 75 years (range 45-93 years), and 159 (82%) were males. Nine (5%) of these patients had already been diagnosed with dementia. The median number of days from the date of the Mini-Cog test in the preoperative clinic to TURBT was 15 days (interquartile range: 9-23).

Based on the Mini-Cog test, the patients were dichotomized into 2 groups: 37 (19%) patients had probable cognitive impairment (Mini-Cog scores < 3). Postoperative delirium occurred in 24 patients (12%).

### Association Between the Mini-Cog Test and Clinical Variables

Baseline and perioperative characteristics of the study population, stratified by Mini-Cog test are shown in Table 1. There were statistically significant differences in patients’ age (*P* < .001), ECOG-PS (*P* = .01), decline in IADL from baseline (*P* = .03), preoperative diagnosis of dementia (*P* < .001), and use of benzodiazepine (*P* = .03) between the Mini-Cog score ≥ 3 group and the Mini-Cog score < 3 group.

### Impact of the Mini-Cog Test for Predicting Postoperative Delirium

Of the clinical factors in the included patients, ECOG-PS ≥ 2 (*P* = .003), IADL decline (*P* = .008), preoperative dementia diagnosis (*P* = .01), preoperative benzodiazepine use (*P* = .01), a Mini-Cog score < 3 (*P* < .001), and bladder neck involvement of the tumor (*P* = .03) were significantly associated with postoperative delirium in the univariate analysis (Table 2). In the multivariate analysis, a Mini-Cog score < 3 (OR = 6.8; 95% CI = 2.7-17.2; *P* < .001) and IADL decline (OR = 3.0; 95% CI = 1.2-7.9; *P* = .02) were independent risk factors for postoperative delirium (Table 2).

## Discussion

The present study demonstrated, for the first time, the clinical significance of a Mini-Cog score < 3 and IADL decline for predicting postoperative delirium after TURBT under awake spinal anesthesia.

The Mini-Cog test is a simple tool for screening cognitive deficits. It may provide a preoperative indication of cognitive decline in patients who have not yet been diagnosed with dementia. In fact, probable cognitive impairment based on the Mini-Cog test was seen in 37 patients (19%) in this study; however, only 9 patients (5%) were previously diagnosed with dementia. The Mini-Cog test has been validated in various clinical settings and is shown to have a high sensitivity and specificity.^9^ In addition, the diagnostic value of the Mini-Cog test is less susceptible to errors caused by the level of education or language of the patient than alternative assessments. For example, the Cognitive Abilities Screening Instrument (CASI) is adversely influenced by low education, and both education level and language ability can compromise the diagnostic value of the Mini-Mental State Exam (MMSE).^4^ The Mini-Cog test is a useful screening test in various settings due to its correlation with the incidence of delirium during post-anesthetic care^7^ and with postoperative delirium after general anesthesia during elective surgery.^8^

In this study, the overall incidence of postoperative delirium was 12% (24 cases), which is slightly lower than the commonly reported incidence of postoperative delirium.^16^ This may be because TURBT is less invasive than other surgeries, and all patients in this study underwent surgery with spinal anesthesia while awake. Multivariate analysis based on the result of delirium incidence revealed that a Mini-Cog score < 3 was significantly associated with postoperative delirium. However, IADL decline was also associated with the occurrence of postoperative delirium. It has been reported that a decline of preoperative IADL is associated with the incidence of postoperative delirium and complications, and the present report shows a similar trend.^17^ Instrumental activities of daily living is more complex than the activities of daily living (ADL) needed for basic unassisted living and requires higher-order functions for elders. Therefore, maintaining ADL, especially IADL for elderly patients, may be important in postoperative management. In particular, postoperative delirium after transurethral surgery may lead to accidental Foley catheter removal, which might result in catheter-associated urinary tract infections and, although rare, life-threatening hematuria. As we demonstrated, a formal, yet simple and brief, cognitive screening procedure can be useful both to identify probable cognitive impairment before surgery and, in conjunction with other information gathered routinely preoperatively, to forecast which patients are most likely to have undesirable postoperative outcomes. Moreover, most subjects endorsed the use of a brief memory test preoperatively. Therefore, it was suggested that preoperative assessment of cognitive function using the Mini-Cog test may contribute to more appropriate patient management after TURBT surgery. 

Although the screening of cognitive function using the Mini-Cog test has been useful in predicting the occurrence of delirium after general anesthesia,^7,8^ this is the first report to show the usefulness of the Mini-Cog test in predicting postoperative delirium after surgery under spinal anesthesia in the awake state. Regardless of the anesthesia method, perioperative management that reduces the stress of invasive procedures will be required for patients with cognitive impairment. For example, the timing of cessation of bed rest can be accelerated, or prophylactic pain medication can be used before complaints of pain arise.

Our findings had several limitations. Although the sample size and number of cognitively impaired patients in this study were not smaller than in previous studies,^4,7,8^ the data were collected at a single institution. Therefore, the results may not be generalizable to other institutions or patient populations. Besides, because our institution is a cancer center, only patients who underwent TURBT were included in the study, and other urological surgeries under spinal anesthesia such as transurethral resection of the prostate and transurethral lithotripsy were not examined. Further studies from other centers are required to validate our findings. Another limitation of this study was that the determination of postoperative delirium was made by the attending physician based on the *DSM-5*. Delirium was not determined using a highly objective index such as the Confusion Assessment Method. Qualitative diagnosis of delirium may be predisposed to errors compared to objective assessments. Similarly, the presence of preoperative dementia was determined only by interviewing the family and consulting the patient’s medical history and not by objective indicators. Finally, we did not preoperatively evaluate cognitive function with the CASI or MMSE, and we were not able to show the superiority of the Mini-Cog test or a relationship between these measures.

In conclusion, the Mini-Cog test is a simple screening tool with only 2 components (a delayed, 3-word recall task and a clock drawing test). Cognitive function screening using the Mini-Cog test before surgery may help identify patients at risk of postoperative delirium. Effective screening opens the possibility for targeted interventions to reduce the consequences of delirium.

## Figures and Tables

**Table 1.** Baseline and Perioperative Characteristics of the Patient Population Stratified by Mini-Cog Test

Variables	Total (n = 193)	Mini-Cog < 3 (n = 37)	Mini-Cog ≥ 3 (n = 156)	*P*
Male, n (%)	159 (82)	29 (78)	130 (83)	.48
Age, years; median (range)	75 (45 to 93)	80 (49 to 93)	73 (45 to 92)	<.001^***^
ECOG-PS; median (IQR)	0 (0 to 1)	1 (0 to 1)	0 (0 to 1)	.01^*^
CCI; median (IQR)	2 (0 to 2)	2 (1 to 2)	2 (0 to 2)	.38
Decline in IADL from baseline; median (IQR)	0 (−1 to 0)	−1 (−3 to 0)	0 (−1 to 0)	.03^*^
Dementia diagnosis; n (%)	9 (5)	7 (19)	2 (1)	<.001^***^
Depression diagnosis; n (%)	3 (2)	2 (5)	1 (1)	.07
Preoperative medications; n (%)				
Steroids	7 (4)	2 (6)	5 (3)	.52
Benzodiazepine	18 (9)	7 (19)	11 (7)	.03^*^
Operative time, minutes; median (IQR)	32 (22 to 51)	29 (19 to 49)	32 (22 to 52)	.50
TURBO; n (%)	18 (9)	4 (11)	14 (9)	.73
Tumor location; n (%)				
Bladder neck	47 (24)	13 (35)	34 (22)	.09
Trigone	59 (31)	15 (41)	44 (28)	.15
Tumor size, mm; median (IQR)	10 (10 to 20)	10 (10 to 20)	10 (10 to 20)	.85
Number of tumors; n (%)				.36
Single	118 (61)	17 (46)	101 (64)	
2-7	71 (37)	20 (54)	51 (33)	
8 or more	4 (2)	0	4 (3)	
MIBC; n (%)	29 (15)	5 (14)	24 (15)	.77
Intraoperative hypotension; n (%)	25 (13)	6 (16)	19 (12)	.59
Intraoperative bradycardia; n (%)	3 (2)	1 (3)	2 (1)	.47
Intraoperative use of ephedrine; n (%)	11 (6)	3 (8)	8 (5)	.44
Intraoperative use of atropine; n (%)	5 (3)	1 (3)	4 (3)	1.00
Perioperative shivering; n (%)	2 (1)	0	2 (1)	1.00
Postoperative complication, highest; n (%)				.46
CDC 0	170 (88)	32 (86)	138 (88)	
CDC 1	2 (1)	1 (3)	1 (1)	
CDC 2	19 (10)	3 (8)	16 (10)	
CDC 3 or more	2 (1)	0	1 (1)	
Length of hospital stay, days; median (IQR)	4 (3 to 5)	4 (3 to 5)	4 (3 to 5)	.61

Mini-Cog; mini-cognitive assessment instrument, ECOG-PS; Eastern Cooperative Oncology Group-physical status, IQR; interquartile range, CCI; Charlson comorbidity index, IADL; instrumental activities of daily living, TURBO; transurethral resection of bladder tumor in one Piece, MIBC; muscle-invasive bladder cancer, CDC; Clavien–Dindo classification.

**Table 2.** Univariate and Multivariate Analysis of Risk Factors for Postoperative Delirium in 193 Patients who Underwent TURBT

Variables	Univariate Analysis		Multivariate Analysis	
OR (95% CI)	*P*	OR (95% CI)	*P*
Female vs. male	2.2 (0.8-5.7)	.11		
Age; > 65 vs. ≤ 65	3.1 (0.4-24.1)	.26		
ECOG-PS; ≥ 2 vs. < 2	8.6 (2.3-32.6)	.003^**^		
CCI; ≥ 2 vs. < 2	1.4 (0.6-3.4)	.42		
IADL decline; yes vs. no	3.3 (1.3-8.1)	.008^**^	3.0 (1.2-7.9)	.02^*^
Dementia diagnosis; yes vs. no	6.6 (1.6-26.5)	.01^*^		
Depression diagnosis; no vs. yes	3.6 (0.3-41.6)	.34		
Preoperative steroid medication; yes vs. no	3.0 (0.5-16.3)	.24		
Preoperative benzodiazepine medication; yes vs. no	4.4 (1.5-13.0)	.01^*^		
Mini-Cog score; < 3 vs. ≥ 3	7.1 (2.9-17.8)	<.001^***^	6.8 (2.7-17.2)	<.001^***^
Operative time (minutes); < 30 vs. ≥ 30	1.1 (0.4-2.5)	.89		
Conventional TURBT vs. TURBO	2.6 (0.3-20.4)	.31		
Bladder neck involvement; yes vs. no	2.5 (1.0-6.2)	.03^*^		
Trigone involvement; yes vs. no	2.0 (0.8-4.8)	.11		
Tumor size (mm); ≥ 30 vs. < 30	1.1 (0.4-3.1)	.89		
Number of tumors; multiple vs. single	2.0 (0.9-4.8)	.12		
MIBC or not	1.2 (0.4-3.7)	.81		
Intraoperative use of ephedrine; yes vs. no	1.6 (0.3-8.0)	.63		
Intraoperative use of atropine; no vs. yes	1.7 (0.1-30.6)	1.00		
Postoperative complications (CDC, highest); ≥ 3 vs. < 3	7.3 (0.4-120.8)	.19		

OR, odds ratio; ECOG-PS, Eastern Cooperative Oncology Group-physical status; CCI, Charlson comorbidity index; IADL, instrumental activities of daily living; Mini-Cog, mini-cognitive assessment instrument; TURBT, transurethral resection of bladder tumor; TURBO, transurethral resection of bladder tumor in one piece; MIBC, muscle-invasive bladder cancer; CDC, Clavien–Dindo classification.

## References

[1] Siegel RL Miller KD Jemal A . Cancer statistics, 2019. CA Cancer J Clin. 2019;69(1):7 34. 10.3322/caac.21551) 30620402

[2] Brummel NE Girard TD . Preventing delirium in the intensive care unit. Crit Care Clin. 2013;29(1):51 65. 10.1016/j.ccc.2012.10.007) 23182527 PMC3508697

[3] Liang LM Xue J Erturk E . Perineal pseudoaneurysm from traumatic foley removal leads to recurrent life-threatening hematuria. J Endourol Case Rep. 2015;1(1):50 51. 10.1089/cren.2015.0009) 27579388 PMC4996550

[4] Borson S Scanlan J Brush M Vitaliano P Dokmak A . The mini-cog: a cognitive ‘vital signs’ measure for dementia screening in multi-lingual elderly. Int J Geriatr Psychiatry. 2000;15(11):1021 1027. 10.1002/1099-1166(200011)15:11<1021::aid-gps234>3.0.co;2-6) 11113982

[5] Steenland NK Auman CM Patel PM et al. Development of a rapid screening instrument for mild cognitive impairment and undiagnosed dementia. J Alzheimers Dis. 2008;15(3):419 427. 10.3233/jad-2008-15308) 18997295 PMC2679370

[6] Borson S Mini-cog. Instructions for administration & Scoring; 2020. Available at: http://mini-cog.com/wp-content/uploads/2015/12/Universal-Mini-Cog-Form-011916.pdf. Accessed May 2021.

[7] Tiwary N Treggiari MM Yanez ND et al. Agreement between the mini-cog in the preoperative clinic and on the day of surgery and association with postanesthesia care unit delirium: a cohort study of cognitive screening in older adults. Anesth Analg. 2021;132(4):1112 1119. 10.1213/ANE.0000000000005197) 33002933

[8] Dworkin A Lee DS An AR Goodlin SJ . A simple tool to predict development of delirium After elective surgery. J Am Geriatr Soc. 2016;64(11):e149 e153. 10.1111/jgs.14428) 27650453

[9] Tsoi KK Chan JY Hirai HW Wong SY Kwok TC . Cognitive tests to detect dementia: a systematic review and meta-analysis. JAMA Intern Med. 2015;175(9):1450 1458. 10.1001/jamainternmed.2015.2152) 26052687

[10] Ishizaki T Kai I Kobayashi Y Imanaka Y . Functional transitions and active life expectancy for older Japanese living in a community. Arch Gerontol Geriatr. 2002;35(2):107 120. 10.1016/s0167-4943(02)00002-x) 14764349

[11] Kiyoshige E Kabayama M Gondo Y et al. Age group differences in association between IADL decline and depressive symptoms in community-dwelling elderly. BMC Geriatr. 2019;19(1):309. 10.1186/s12877-019-1333-6) PMC6854629 31722665

[12] Hara T Matsuyama H Kamiryo Y et al. Use of preoperative performance status and hemoglobin concentration to predict overall survival for patients aged ≥75 years after radical cystectomy for treatment of bladder cancer. Int J Clin Oncol. 2016;21(1):139 147. 10.1007/s10147-015-0857-9) 26077140

[13] Palumbo C Knipper S Pecoraro A et al. Patient frailty predicts worse perioperative outcomes and higher cost after radical cystectomy. Surg Oncol. 2020;32:8 13. 10.1016/j.suronc.2019.10.014) 31683158

[14] Zhang X Wang J An XH et al. Optimum dose of spinal ropivacaine with or without single intravenous bolus of S-ketamine during elective cesarean delivery: a randomized, double-blind, sequential dose-finding study. BMC Preg Childbirth. 2021;21(1):746. 10.1186/s12884-021-04229-y) PMC8567718 34736438

[15] American Psychiatric Association. Anxiety disorders. In: Diagnostic and Statistical Manual of Mental Disorders. 5th ed . Washington, DC: Author; 2013. 10.1176/appi.books.9780890425596.dsm05)

[16] Inouye SK Westendorp RGJ Saczynski JS . Delirium in elderly people. Lancet. 2014;383(9920):911 922. 10.1016/S0140-6736(13)60688-1) 23992774 PMC4120864

[17] Dogrul RT Dogrul AB Konan A et al. Does preoperative comprehensive geriatric assessment and frailty predict postoperative complications? World J Surg. 2020;44(11):3729 3736. 10.1007/s00268-020-05715-8) 32737555

